# Supplementary material for: Are carbon emissions trading and green financial instruments synergistic? -Comprehensive quantitative research based on content analysis
Source: PLoS One. 2024 Mar 7;19(3):e0298601. doi: 10.1371/journal.pone.0298601 (PMC10919601; doi:10.1371/journal.pone.0298601)
Supplement: S1 Data — (ZIP) [file pone.0298601.s001.zip › Data/地级市Z_F工作报告.zip!/║╙─╧/╚2├┼╧┐╩╨/└┤╫╘╣1⁄2╓┌║┼í╛┬φ┐╦╩2╛▌═°í┐_2019.docx]

各位代表：

　　现在，我代表市人民政府，向大会报告工作，请予审议，并请各位政协委员和其他列席人士提出意见。

　　一、2018年工作回顾

　　2018年，面对复杂多变的国际国内环境和艰巨繁重的改革发展任务，我们坚持以习近平新时代中国特色社会主义思想为指导，全面贯彻党的十九大和十九届二中、三中全会精神，强化“四个意识”，坚定“四个自信”，做到“两个维护”，在省委、省政府和市委的坚强领导下，在市人大、市政协的监督支持下，团结和依靠全市人民，围绕建设省际区域中心城市这一目标，砥砺奋进，迎难而上，经济社会发展保持了稳中有进、稳中提质、稳中向好的态势，圆满完成市七届人大三次会议确定的目标任务。全市生产总值完成1528.1亿元，同比增长8%，人均生产总值10169美元；财政一般公共预算收入120.2亿元，增长11.1%；规上工业增加值增长8%，高于全省0.8个百分点；固定资产投资增长10.5%，高于全省2.4个百分点；社会消费品零售总额增长10.7%，高于全省0.4个百分点；进出口总值增长38.9%，高于全省33.6个百分点；民间投资增长18.2%，高于全省15.3个百分点。今年一季度，全市生产总值增长8.4%，增速居全省第6位；财政一般公共预算收入增长22.9%，居全省第3位；规上工业增加值增长9.4%，居全省第4位；固定资产投资增长8.5%；社会消费品零售总额增长10.9%，居全省第4位，进出口总值增长32%，居全省第4位，主要经济指标位居全省第一方阵。全市经济社会发展迈出了高质量发展的新步伐，交出了一份逆势前行、强劲发展的年度答卷! 主要呈现出四个特点：

　　——经济社会发展实现了“三个同步、三个高于”。生产总值、财政收入和居民收入增速分别高于全省0.4、0.6和0.1个百分点。居民收入与生产总值同步增长，居民人均可支配收入增长9%，增速高于生产总值1个百分点。生态环境质量与经济质量效益同步改善，PM2.5年累计平均浓度完成省定目标，空气优良天数达到212天，优良率63.3%，是全省唯一一个超额完成目标任务的省辖市。社会事业进步与经济发展水平同步提高，累计完成民生财政支出179.3亿元，增长13.7%，占财政一般公共预算支出的73.5%，新增义务教育标准学位4320个，职业教育园区建设扎实推进，大专新招生2100名，市妇女儿童医院主体完工，教育、医疗、文化等社会事业全面进步。

　　——重大攻坚成效显著。易地扶贫搬迁提前两年完成“十三五”搬迁任务，全国现场会到我市观摩，全省现场会在我市召开。金融扶贫“三项机制”在国务院第五次大督查中作为全国唯一的金融扶贫典型案例受到通报表彰。黄河湿地国家级自然保护区年度生态修复任务全面完成，小秦岭自然保护区在“绿盾2018”国家专项检查行动中受到充分肯定。全面开展黄河河道专项清理整顿，基本解决了困扰黄河河道几十年的“老大难”问题。投资集团融资能力持续提升，信用评级达到AA+，全年直接投资44.7亿元，带动全社会投资365.6亿元。

　　——重大项目实现突破。速达10万辆纯电动汽车项目通过工信部技术审查，成为全国第11家、河南省首家获得“双认证”资质的企业，量产首车正式下线，实现全省本土品牌基本乘用轿车和纯电动汽车“双突破”。金城冶金项目建成投产，成为全国冶金行业标杆企业。国道310南移项目实现投资、进度“双过半”；蒙华铁路营运中心顺利落户三门峡，将有力促进货运枢纽建设，打造全国重要物流基地。同人铝业与宝武集团签订合资合作协议，铝工业向中高端延伸迈出关键步伐。新争取的全国第二家进口铜精矿检验检疫试验区基本建成，将建起“家门口的海外原料基地”。高阳山大健康产业园入选省级现代服务业专业园区。新型智慧城市“互联网+政务服务” 走在全省前列、“12345”便民服务热线运行模式全省推广，受到中央、省委领导的肯定和鼓励！

　　——重大活动圆满成功。第二十四届黄河文化旅游节签约经济合作项目85个，总投资465.4亿元，会展经济带动效应持续放大。成功举办第十二届中国摄影艺术节，参展摄影家、观展人数、网上点击量均创历届之最，我市被授予“中国摄影之乡”称号。在此基础上，乘胜追击、扩大战果，成功争取中国摄影艺术节第13至17届落户，并与中摄协开展全面深度合作，在三门峡谋划建设集“产学研游展”为一体的中国摄影文化城，打造全国摄影艺术文化高地。“魅力中国城”活动效应持续显现，被评为首批全国生态旅游胜地，天鹅湖湿地公园上榜“年度魅力生态景区”，陕州地坑院上榜“年度魅力乡村旅游目的地”。

　　一年来，我们主要抓了以下工作：

　　（一）集中力量克难攻坚，坚决打好三大攻坚战。把打好三大攻坚战作为重大政治任务和重大发展机遇，动员全市力量，采取超常规举措，持续向纵深推进。防范化解重大风险。坚持把防范化解重大风险作为底线工程，出台专项方案，化解政府债务风险，全市综合债务率和债务规模稳定在全省中低水平。健全金融风险联防机制，开展非法集资风险专项排查，一批问题企业、问题项目、问题楼盘得到有效处置，守住了不发生区域性金融风险的底线。强化联防联控，非洲猪瘟实现疫情零发生。扎实开展精准脱贫。坚持精准方略，聚焦深度贫困，打好产业扶贫等“五大硬仗”，实施健康扶贫等“七大行动”，实现53733人脱贫，103个贫困村稳定退出，全市贫困发生率由2017年底的5.46%下降到2018年底的2.22%。强力推进污染防治。积极实施污染防治攻坚战三年行动计划，强化“六控”推进源头预防、调整“四个结构”加强综合治理、精准发力实施重点管控，空气环境质量连续两年位居全省前列。深入推行河、湖长制，扎实开展河道采砂综合治理、黑臭水体歼灭战，全面启动“四水同治”和国土绿化行动，环境承载力持续提升。

　　（二）着力稳定经济运行，夯实高质量发展基础。坚持把推动经济平稳增长作为全局工作的首要任务，及时分析研判，加强运行调度，解决突出问题，为高质量发展创造良好条件。着力扩投资。18个省重点项目完成投资121.5亿元，占年度目标的107%；221个市重点项目完成投资720亿元，占年度目标的125%，中金二期等46个项目建成投产，经济活力持续迸发。加快 “去降补”。化解过剩煤炭产能192万吨，房地产去库存周期保持在8个月，出台8个方面58条降成本措施，新增电力直接交易用户220户，累计降低用电成本9227万元。突出抓保障。深入开展“精准服务企业、助力转型发展”活动，突出抓好100家重点企业生产经营情况监测分析，深化“四项对接”，破解瓶颈制约。积极组织银企对接，117个项目达成合作协议，授信额度443.7亿元。持续强载体。产业集聚区和服务业“两区”建设迈出新步伐，三门峡产业集聚区、渑池县产业集聚区晋升二星级，湖滨区特色商业区晋升二星级，市商务中心区、渑池县、陕州区特色商业区晋升一星级，带动作用不断增强。

　　（三）持续打好“四张牌”，推动经济高质量发展。坚持把打好“四张牌”作为落实新发展理念、深化供给侧结构性改革、推进高质量发展的关键举措和抓手。持续推动产业结构优化升级。推动工业加快转型，实施3000万元以上工业转型升级项目111个，完成投资250亿元，宝鑫电子等28个项目建成投产。强力推进“三大改造”，入库项目160个，建成开祥化工、骏通车辆、戴卡轮毂等一批绿色工厂、智能工厂和智能车间。推动服务业提档升级，天鹅湖国家级旅游度假区建设扎实推进，双龙湾旅游度假区成功创建省级旅游度假区，大一物流实现我市A级物流企业零突破，跨境电商交易额突破50亿元，同比增长50%。推动农业提质增效，陕州区成功创建国家级农村一二三产业融合发展示范园，二仙坡果业荣获国家级农业产业化重点龙头企业，实现全市零突破。新认定省级示范性产业化联合体7家，省级农业产业化集群10家，新发展家庭农场30个，农业经营主体更加完善。涉农电商交易额突破50亿元。全市第一产业增加值增长4.1%，继续保持全省第一位。大力实施创新驱动发展战略。激活科技创新第一动力，国家级高新区创建通过科技部现场验收，新增高新技术企业26家，实现历史最快增长，新认定省级企业技术研发中心24家，科技创新能力稳步提升。多层次资本市场建设高效推进，新增区域性股权市场挂牌企业134家。落实人才引进“1+8”系列政策，引进创新创业领军人才（团队）52个，扶持人才项目38个，总投资15亿元。积极推进新型城镇化。《百年崤函—三门峡2057远景发展战略》编制完成，百城建设提质工程和文明城市创建扎实推进，中心城区新增供热面积170万平方米，庙底沟遗址公园基本建成，甘棠路大桥通车在即，城市综合承载力持续提升。加快县（市）城区基础设施扩容提质，谋划实施重点项目328个，总投资550亿元，县（市）城区面貌明显改观。大力开展农村环境综合整治，创建“四美乡村”108个，“四美乡村”示范乡镇2个，“五美庭院”10732个。农村“厕所革命”、“大棚房”专项整治扎实推进。不断强化基础能力建设。三淅高速灵宝段具备通车条件，黄河公铁两用桥、渑垣高速项目稳步推进，交通枢纽地位更加牢固。大石涧水库主体完成，张家坡水库、池芦水库基本建成，完成农村危房改造2616户，新改建农村公路487公里，解决26.6万农村居民饮水安全问题，农村生产生活条件持续改善。

　　（四）继续深化改革开放，不断增强高质量发展活力。充分用好改革开放“关键一招”，聚合改革正能量，拓展开放新优势，为实现高质量发展注入强劲动能。深化重点领域改革。全面打响国企改革“总攻战”，提前3个月完成省定52家“僵尸企业”处置任务，被省政府评为国企改革攻坚先进市。“放管服”改革深入推进，897项事项列入“最多跑一次”清单，969项实现“一网通办”，累计网上办件28.5万件，群众满意率达到99.8%。新型智慧城市框架基本建立，“两平台、三中心、十应用”建成运行。农村土地确权颁证完成，集体产权制度暨“三变”改革全面启动。政府机构改革任务基本完成，财税、盐业、投融资、社会信用、电力体制等改革稳步推进。持续扩大开放合作。围绕十大重点产业开展对口招商，全年签约项目136个，总投资884.7亿元，其中10亿元以上项目25个。在首届中国国际进口博览会上签约26.5亿元，居全省第1位。加强与西安市对接，在科技、教育、医疗、人才等10个领域深入合作。

　　（五）坚持以人为本，让人民共享高质量发展成果。坚持以人民为中心的发展思想，持续扩大投入，抓好民生实事，不断增强群众获得感、幸福感和满意度。扎实推进就业创业。坚持就业优先战略，发放创业担保贷款4.5亿元，带动就业1.04万人，城镇新增就业4.74万人，城镇登记失业率2.77%。兜牢民生保障底线。退休人员养老金、城乡低保标准持续增长，困难群众大病补充医保、困难残疾人两项补贴全面落实，全民参保计划、健康养老“十百千”示范项目等扎实推进，48个棚改项目全部开工，社会保障体系更加完善。加快发展民生事业。坚持教育优先发展，成功引进东方剑桥等优质教育资源，完成新改扩建中小学35所、幼儿园19所，6个县（市、区）全部通过“全国义务教育发展基本均衡县”验收，职业技术学院新校区建设全面启动。加快“健康三门峡”建设，黄河医院商务中心区分院主体封顶，与北京301医院初步达成共建华中消化病医院协议，市中心医院综合实力跨入全省第一梯队，区域医疗水平不断提升。扎实推进公共文化服务体系建设，全面启动 “仰韶文化发现100周年”纪念活动，市博物馆晋升国家二级馆，《大山的儿子》作为河南省唯一代表剧目进京展演。积极创新社会治理。深入开展扫黑除恶专项斗争，积极预防和化解社会矛盾，安全生产、食品安全和信访形势平稳，全市公众安全感位居全省第1位，社会大局和谐稳定。

　　在做好各项工作的同时，我们狠抓政府自身建设，始终坚持以政治建设为统领，在政治上思想上工作上与上级全面对标对表，坚决贯彻落实党中央国务院、省委省政府和市委的各项重大决策部署，确保全市经济社会发展与党中央要求步调一致。落实全面从严治党主体责任，深入推进政府系统党风廉政建设，积极开展中央、省委巡视反馈问题整改，充分发挥审计监督作用，自觉接受人大、政协和社会监督，认真办理市人大代表建议152件、政协提案265件。加强国防教育、国防后备力量和人民防空建设，推进军民融合深度发展，驻峡解放军、武警部队、预备役人员、民兵为地方发展作出新贡献，双拥工作持续深化。统计、外侨、民族宗教、对台、史志、气象、防震减灾、无线电、黄河河务、妇女儿童、残疾人、老龄、慈善等工作取得新成绩。

　　过去的一年，面对挑战和考验，我们以坚如磐石的决心和毅力 ，在克难攻坚中铿锵前行，在实现中原更加出彩的新征程中书写了三门峡的新篇章。在实践中我们深切体会到：第一，必须加强领导，把握全局。党的集中统一领导是中国特色社会主义制度的最大优势，是统筹力量、集思广益、科学谋划的最有效制度安排。我们深入落实中央、省委和市委决策部署，一方面“强身健体”，大力发展有特色的高端制造业、现代服务业、高山农业等产业集群，扎实推进“三地五中心”建设，不断增强经济活力和区域核心竞争力；另一方面“凝神聚力”，关注百姓需求，认真解决“七大难”等民生问题，群众获得感、幸福感持续提升，不断巩固执政基础，全市呈现出心齐气足、奋发向上的新气象。第二，必须迎难而上，抢抓机遇。面对转型创新的繁重任务和区域竞争更加激烈的严峻形势，危险与机遇相伴共生，我们始终保持清醒坚定，保持定力耐力，敢于虎口拔牙、事不避难，担责担难担险，坚忍不拔，久久为功，促成了速达电动汽车、宝武铝业、金城冶炼、310国道改线、蒙华铁路营运中心、铜精矿国检试验区等一批具有战略支撑意义的重大项目落地，拓展了发展的新空间，培育了发展的新动能。第三，必须解放思想，勇于创新。我们主动顺应IT向DT转变的时代变革，积极适应发展竞争越来越“人才化、年轻化”的新趋势，解放思想，超前谋划启动了新型智慧城市建设和职业教育园区发展，闯出了政府管理创新和城市转型发展的新路，加快了新型工业化城市、高成长服务业先进市、特色农业强市、网络经济大市建设步伐。第四，必须团结奋进，聚力聚势。团结是黏合剂，团结是催化剂，团结出生产力，团结出战斗力。近几年我市出现的发展好局面、发展好势头，得益于全市上下齐心协力，得益于广大干群众志成城。众人划桨开大船。只要我们一如既往和衷共济、聚心聚神、聚力聚势，团结一切可以团结的力量，发挥一切积极有利因素，就一定能够克服前进中的一切困难，战胜一切艰难险阻，巩固发展好形势，谱写高质量发展新篇章！

　　各位代表！过去一年取得的成绩，是习近平新时代中国特色社会主义思想科学指引的结果，是省委、省政府和市委正确领导的结果，是全市人民凝心聚力，埋头苦干的结果，是各级各部门尽职尽责，顽强拼搏的结果。在此，我代表市人民政府，向全市各行各业的劳动者，向各位人大代表、政协委员，向各民主党派和人民团体，向驻峡解放军、武警官兵、公安干警，向在三门峡投资创业以及关心支持三门峡发展的各界人士，致以崇高的敬意和衷心的感谢！

　　思危方能居安。我们清醒认识到，我市发展不平衡不充分问题还比较突出，工作还存在不少挑战和薄弱环节。主要表现为：一是转型发展任务艰巨。增长方式粗放、结构矛盾突出、创新能力不强的状况尚未根本改变，新产业、新业态、新模式依然发育不足，发展新动能活力不够，推动高质量发展任重而道远。二是部分企业经营困难。经济下行压力增大，市场需求增长放缓，企业融资困难加剧，综合成本上涨较快，部分企业经营困难，利润下滑较多。三是民生领域仍有短板。城市建设方面欠账较多，新老城区发展不平衡的问题比较突出，棚户区改造任务繁重，“七大难”解决的标准不高，安全生产、公共安全等领域依然存在薄弱环节，社会风险隐患不容忽视。同时，政府职能转变还不到位，一些公务人员综合素质还不适应新时代要求，“四风”问题仍然存在。对此，我们一定高度重视，切实解决，决不辜负人民重托。

　　二、2019年工作主要目标和重点任务

　　2019年是新中国成立70周年，是决胜全面建成小康社会、实现第一个百年奋斗目标的关键一年，做好今年的工作意义重大。今年政府工作要认真落实市委七届七次全会要求：以习近平新时代中国特色社会主义思想为指导，全面贯彻习近平总书记视察指导河南时的重要讲话精神和中央经济工作会议精神，认真落实省委十届八次全会部署和省委主要领导调研指导三门峡时的讲话要求，坚持稳中求进工作总基调，坚持新发展理念，以供给侧结构性改革为主线，贯彻巩固、增强、提升、畅通的方针，聚焦高质量建设省际区域中心城市，打造“五彩三门峡”、建设“三地五中心”，发力“三次创业”，加快转型创新发展，深化“五个努力”，进一步稳就业、稳金融、稳外贸、稳外资、稳投资、稳预期，保持经济持续健康发展和社会大局稳定，为全面建成小康社会收官打下决定性基础，以优异成绩庆祝中华人民共和国成立70周年。

　　经济社会发展主要预期目标是：生产总值增长7.5%—8%，努力在工作中取得更好的成果；规上工业增加值增长8%，第三产业增加值增长9%，全员劳动生产率增长7%，一般公共预算收入增长9%，税收占一般公共预算收入的比重达到72%以上，固定资产投资增长9%左右，社会消费品零售总额增长10%以上，居民消费价格涨幅控制在3%左右，经济结构进一步优化，质量效益明显提升。研究与试验发展经费投入强度达到1.3%；常住人口城镇化率提高1.3个百分点以上；单位生产总值能耗和主要污染物排放量完成省下达目标；进出口保持平稳增长；居民人均可支配收入增长8.5%，城镇登记失业率保持在3.5%以内。

　　根据总体要求和发展目标，我们把推动经济平稳增长作为全局工作的首要任务，确定了“1291”工作思路。即：紧紧围绕高质量建设省际区域中心城市这一定位，加快城市和产业两大转型，聚焦九项重点工作，确保经济实现平稳健康增长，主要经济指标保持全省第一方阵。重点抓好以下工作：

　　（一）聚焦稳定经济增长，巩固稳中有进发展态势，实现经济平稳健康发展。坚持稳中求进工作总基调，贯彻落实好“八字方针”，突出供给侧结构性改革这一主线，补短板、扩内需、强实体、促转型，确保经济行稳致远。

　　保持工业稳定增长。紧紧稳住工业这个“定盘星”，切实加强运行调度和监测分析，强化要素保障，优化企业服务，确保工业经济平稳运行。把推动制造业高质量发展作为稳工业的重要抓手，大力实施“三大改造”，扎实开展智能制造引领、重点技术攻坚、传统产业提质、新兴产业培育四大行动，重点支持主营业务收入20亿元以上大型制造业企业、1亿元以上高成长制造业企业发展，加快推进宝武铝业等一批重大项目建设，夯实产业发展基础。

　　全面实施“十大工程”。围绕强基础、补短板、增活力，全力推进棚户区改造、国土绿化与“四水同治”、决战脱贫攻坚、农村人居环境与城乡结合部整治、优化营商环境、工业“三大改造”与盘活企业存量、职业教育园区建设、金融创新、点亮天鹅城、交通建设等“十大工程”，集中精力，重点攻坚，确保45个重大项目完成年度投资230亿元，以重大工程的落地见效促进2019年各项工作目标实现，全面提升经济发展质量效益。

　　加大招商引资和项目建设力度。坚持把开放招商作为动力之源，积极对接省“六大项目库”，深入推进全链条、集群化招商，用好“专人专班”工作机制，着力在高端制造、现代服务业等产业集群招商上实现新突破。坚持把抓好重大项目作为落实

　　“十大工程”的具体抓手，继续完善领导分包、联审联批、督导考核、月度协调等推进机制，重点抓好总投资1809亿元的215个重点项目，确保年度完成投资620亿元以上。

　　突出抓好优化营商环境。营商环境就是生产力、创新力、竞争力，要以打造全省先进和中西部地区营商环境一流城市为目标，全面开展“优化营商环境工作年”活动，聚焦营商环境核心指标，努力推动政务服务、企业创办、不动产登记、纳税便利度、跨境贸易等指标达到或接近国内先进地区水平，让一流营商环境成为我市的新标识。坚持“两个毫不动摇”，积极落实国家减税降费政策，大力支持民营经济和中小微企业发展，探索建立非公有制经济产业园，建立市级领导分包服务民营企业制度，构建“亲”“清”新型政商关系，让企业家放手发展、放心发展。大力弘扬企业家精神，培育壮大民营企业家队伍，推动民营经济“个转企”“微升小”“小升规”“规改股”“股上市”，加快形成“中小企业铺天盖地、大型企业顶天立地”的新局面。

　　（二）聚焦打赢打好三大攻坚战，抓重点补短板强弱项，突破全面建成小康社会的重大关口。把打好三大攻坚战摆在全局突出位置，打好重点战役，确保再战再捷，交出历史新答卷。

　　决战决胜脱贫攻坚。进一步提高政治站位，尽锐出战，精准施策，全力支持卢氏县高质量脱贫摘帽，完成全市2.55万人脱贫、181个贫困村退出任务。提高脱贫质量。聚焦“两不愁，三保障”，继续打好“五大硬仗”、开展“七大行动”、实施“四大工程”，巩固“百千万”工程成效，拓展优化金融扶贫“三项机制”，持续提升产业扶贫质量，增强内生动力，实现贫困群众稳定脱贫、可持续发展。聚焦特殊贫困群体。积极探索政府投入、“三变”筹资、社会捐赠等筹资渠道，对五类特殊贫困群体实施集中供养、集中治疗、妥善安置，保证他们生存有保障、生活有尊严。加强易地搬迁后续帮扶管理。持续加强产业配套、公共服务配套和就业安置，全面推进易地扶贫搬迁美好生活“5个新”专项活动，深入实施公共服务“5个有”覆盖工程，从根本上解决搬迁贫困人口的稳定脱贫和持续发展问题。切实搞好统筹推进。加大智志双扶力度，激发贫困群众内生动力。抓好防止贫困人口返贫工作。加大对非贫困县、非贫困村和非贫困人口的政策支持力度，统筹做好贫困“边缘户”的帮扶工作。

　　持之以恒抓好污染防治。深入践行习近平生态文明思想，扭住改善生态环境质量这个核心，坚持污染减排和生态扩容两手发力，扎实做好中央、省环保督察反馈问题整改，以环境质量的明显改善回应人民群众的热切期盼。打好蓝天保卫战。突出重点区域、重点行业、重点污染物，加快产业、能源、运输、国土四大结构调整，持续抓好“六控”，突出治理“两散”，着力解决好结构性、季节性、区域性污染问题，努力让老百姓享受更多的蓝天白云好空气。打好碧水保卫战。持续做好城市“水文章”，加大水源地保护和水源涵养力度，“一河一策”精准治污，多管齐下推进减量排放、截污纳管、面源控制、生态净化，不断改善水环境质量，还老百姓水清岸绿、鱼翔浅底的景象。打好净土保卫战。强化土壤环境安全管控，扎实做好土壤污染防治、工业固废安全利用等工作，推进受污染耕地的安全利用和治理修复，努力守护一方净土。推进生态扩容。高标准开展国土绿化，完成植树造林32.1万亩，森林抚育52.6万亩，廊道绿化4.5万亩，矿山修复6800亩，打造国土绿化、美化先进市和样板市。

　　稳妥防范化解重大风险。紧盯重点领域，解决突出问题，坚决把风险管住，把底线守住。防控政府债务风险，坚持化解存量与严控增量并重，依法规范政府举债行为，坚决遏制变相举债和隐性负债，确保政府债务安全可控。防范金融风险，完善金融监管体系，严厉打击非法集资、非法互联网金融服务、恶意逃废银行债务等行为，持续优化金融生态环境，牢牢守住不发生区域性金融风险的底线。化解企业风险，加强企业风险排查管控，综合施策降低企业资金风险，妥善处理企业担保链、资金链风险。积极推进市场化“债转股”，引导企业加强债务管理，实现稳健有序发展。

　　（三）聚焦转型升级，加快发展特色经济和培育战略性新兴产业，持续增强发展动能。紧紧围绕打造“三地五中心”，培育区域核心竞争力，持续优化结构和产业产品，超前谋划布局一批重量级未来产业，推进一批重点项目、龙头企业和示范工程，备足高质量发展后劲。

　　培育壮大高端制造产业基地。打造新能源汽车产业集群。全力支持速达公司生产经营，确保全年产量不低于2万辆，积极引进电池、电机等上下游配套企业，着力打造以纯电动汽车为主导的研发生产基地、配套加工基地，扎实推进千亿级产业集群建设。打造铝精深加工产业集群。以新材料为引领，积极引进汽车轻量化、航空铝材、汽车轮毂等高端铝加工项目，持续深化与宝武集团、锦江集团等合作，加快推进宝武铝业铝合金铸造、东方希望锻造轮毂、骏通公司铝合金专用车等项目，努力打造中高端铝产品生产基地。打造精细化工产业集群。依托全省少有的义马、陕州两大专业化工园区优势，加快引进一批医药中间体、化工新材料、高端煤化工等前沿化工项目，努力建设全省最大的精细化工产业园区。打造铜精深加工产业集群。依托黄金冶炼伴生50万吨金属铜的产业优势，培育壮大电解铜箔、压延铜箔等产业，建设全国最大的动力电池专用铜箔生产基地，打造“中国铜箔谷”。大力发展电力电子、国防军工、航空航天、汽车船舶等领域铜精深加工产品，把铜产业培育成为工业经济新的增长极。

　　加快提升培育现代服务业产业基地。打造旅游康养产业集群。围绕白天鹅这一独特资源，依托天鹅湖国家城市湿地公园，加快创建国家级旅游度假区，以文化主题公园为载体，启动建设爱情主题小镇和规划建设中国摄影文化城，打造特色旅游新的引爆点，持续擦亮“天鹅之城”城市名片。科学编制大健康产业发展规划，重点抓好高阳山温泉度假区、函谷关历史文化旅游示范区、汤河温泉小镇等项目建设，打造全国新兴的康养旅游目的地。打造现代物流产业集群。充分发挥我市综合交通枢纽和产业基础优势，以蒙华铁路三阳站建设为重点，以保税物流和功能性口岸建设为抓手，积极构建大宗商品、电商、冷链、快递等物流体系，力争铝、苹果等部分大宗商品成为全国价格采集点，重点抓好中欧大宗商品物流园、豫西煤炭储配物流基地等项目建设，全年新增3A级以上物流企业3家以上，加快建设集公铁联运为特色的陆路物流港。打造黄金珠宝加工交易集群。支持中金集团金银制品加工交易项目建设，培育壮大金银制品生产规模，推动黄金产业从生产型制造向服务型制造延伸扩展，打造以设计、生产、销售为一体的黄金珠宝全产业链。

　　（四）聚焦新型城镇化，加快百城建设提质和文明城市创建，提升省际区域中心城市辐射力带动力。加快推进以人为核心的新型城镇化，不断提高城市综合承载力、辐射力，迅速提升首位度、宜居度，建设“疏密有度、高低有序、错落有致、显山露水”的宜居宜业之城，让居民生活更便捷、更舒适。

　　高质量推进城市建设。落实“多规合一”要求，科学编制城市总体规划和专项规划，优化城市空间布局，促进城市发展由外延扩张向内涵提升转变。持续完善城市基础设施，加快国道310南移、职业教育园区等重大工程建设，启动国道209、310城区段和高铁南站提升改造工程，完成黄河路等11条城市道路新改建任务，加快推进中心城区“断头路”打通、污水管网等项目建设，进一步提升城市基础承载能力。启动老旧城区改造工作，坚持“一次规划、分步实施”，宜改则改，宜搬则搬，增加服务功能，留住城市记忆，年内完成项目总体规划及国家专项债申报，启动建设路沿线旧城改造试点，用2—3年时间，实现老旧城区焕然一新。全面启动城市生态水系工程。推动商务中心区南扩西融，实施崤函生态廊道、通秦路东延等项目，争取跨铁路关键性工程早日开工，打通城市环线“快速通道”，促进与陕州区对接融合发展。

　　创新城市经营管理体制机制。把城市作为最大的国有资产，运用市场机制，对城市的土地、河湖等自然生成资本，市政设施等人力作用资本，路桥冠名权、广告设置使用权等相关延利资本进行包装、重组、运营。掌握土地一级市场，统一土地征收、土地储备、土地出库和土地交易，推动土地资源变资产、资产变资本、资本变资金。通过特许经营或转让经营权，盘活文体中心等城市公共设施，实现城市资源配置容量和效益的最大化、最优化。加强市容环境卫生管理，抓好“三无”小区及背街小巷环境整治，建设完善网格化监管体系，提升城市精细化管理水平。

　　确保文明城市创建一举成功。对照省级文明城市创建标准，全力梳理、补齐硬件设施短板，确保6月底前大头落地，9月底前全部达到验收标准。大力推进城市“四治”，广泛激发基层“细胞”活力，加大城市环境、文明交通、违章建筑、户外广告等领域专项整治，全面优化城市环境秩序，实现软硬件双达标，确保创建工作圆满成功。

　　（五）聚焦乡村振兴，着力实现农村特色产业、人居环境和农民收入新提升，开启农业农村现代化新征程。坚持乡村振兴战略总要求，围绕“农业强、农村美、农民富”，加快推进产业、人才、文化、生态、组织“五个振兴”，全面打造乡村振兴示范市。

　　突出抓好特色产业发展。以发展“六优”产品为重点，调整种养、品种、产业结构，努力把资源优势变成经济优势、把经济优势变成产业优势。加快引进农业产业化知名企业，做大做强一批精深加工型农业龙头企业，大力培育发展农业合作社、家庭农场等新型经营主体，持续推进陕州区二仙坡、甘山果香田园综合体、寺河山苹果小镇、卢氏香菇小镇建设，引导和推动更多的生产要素向农业农村流动，保持乡村经济发展旺盛活力。

　　大力实施农村人居环境整治。坚持把改善农村人居环境作为实施乡村振兴的第一场硬仗，认真学习浙江“千村示范、万村整治”经验，以创建“四美乡村”、“五美庭院”、“美丽小镇”为抓手，以交通沿线村庄、旅游景区、易地搬迁社区为重点，持续推进农村人居环境整治三年行动，启动实施“百村示范工程”，重点抓好垃圾和污水处理、“厕所革命”、村容村貌提升，加快建设美丽宜居乡村。

　　持续提高农民收入。扎实推进农村集体产权制度暨“三变”改革，探索农村集体经济新的实现形式和运行机制。积极推动贫困户与带贫企业建立稳定的利益联结机制，让更多贫困群众深度参与产业发展，掌握致富技能，实现稳定增收脱贫。探索建立“省市县+农户”四位一体的风险防控体系，推动普惠金融政策落实落地，引导农业合作社、种植大户、贫困户积极参与特色农产品保险，提高农业抗风险能力。

　　（六）聚焦创新驱动发展，加快建设五大平台，推动新旧动能加速转换。始终把创新摆在发展全局的核心位置，下好创新“先手棋”，全面提升创新能力和效率，着力塑造更多依靠创新驱动的引领型发展。

　　建好科技创新平台。持续推进国家级高新区创建，确保顺利通过国家评审。加力培育科技型创新企业，建好各类科技创新平台，加强科技企业孵化器建设，进一步壮大创新主体，推进构建体系健全、功能完备、开放竞争、富有活力的创新引领平台格局，全年新增国家高新技术企业15家、省级以上创新平台5个，全市科技研发投入不低于1.3%。

　　持续完善开放平台。集中力量壮大通关体系，加快国家级电商示范基地、全国跨境电商服务综合试验区建设，积极申报国家级香菇出口基地，确保河南自贸区三门峡联动发展区挂牌。争取铜精矿检验检疫试验区尽快验收投用，积极创建国家级铜箔产品质检研发中心，全力打造铜箔国家标准。

　　提升产业承接平台。以产业集聚区和服务业“两区”为重点，改进园区管理办法，强化基础支撑，突出服务功能提升、特色集群培育和精品示范引领，确保每个园区固定资产投资、工业增加值分别增长15%、10%以上，突出发展特色，形成产业集群，使之成为我市承接产业转移的有效平台。

　　完善投融资服务平台。支持投资集团做大做强，全年带动投资保持在200亿元以上。加快组建国有资本运营公司、新的专业性产业公司，积极推动农信社改制，组建农村农业水利投资公司，充分发挥好各类基金作用。积极引进域外金融机构落户，持续抓好企业上市工作，推动5个以上优质中小企业进入多层次资本市场。

　　做强人才平台。围绕培育壮大主导产业，继续实施高层次创业创新人才（团队）引进计划、产业人才支撑计划、“名校英才来峡”计划，深化与高校、科研院所合作，提高人才引进成效。

　　（七）聚焦基础能力建设，高水平推进重要基础设施和重大公共服务项目，夯实高质量发展根基。加快交通和水利能源通讯等基础设施建设，破解城乡发展瓶颈，增强发展基础支撑。

　　抓好重大交通项目。围绕区域性交通枢纽建设，全面完善更加高效便捷的现代综合交通体系，实现对外畅通快捷、对内四通八达。对外实施大联通。加快推进渑垣高速、栾卢高速、黄河公铁两用桥南引桥及南引线等在建项目进度，积极配合蒙华铁路建设，确保“十一”顺利通车，争取渑栾高速、连霍呼北高速联络线上半年开工，卢洛高速年内开工。加快推进国道209线城区段改线项目前期工作，力争上半年开工建设，全面提升我市对外联通能力。对内畅通微循环。加快推动陇海铁路取直改线项目前期工作，争取尽快开工，早日消除对城市的分割影响。全面启动甘山、寺河山、燕子山三山联线开发，高水平建设旅游专用公路，打造精品旅游线路。谋划推进卢氏通用机场项目，新建“四好农村路”260公里，提高道路通行能力。

　　加快重大公共服务设施建设。积极推动中国金像艺术馆等中国摄影文化城重要基础平台建设，助推摄影艺术文化新发展。启动实施市民中心、城市规划馆、美术馆、少儿图书馆、融媒体中心等项目，积极推进5G网络建设，完善城市公共服务体系。加快推进新型智慧城市一体化项目，优化提升“两平台”“三中心”“三联网”“十应用”基础支撑能力，推动政务大数据向市大数据中心汇聚，积极发展智慧环保、社会信用系统、120救护平台等特色应用，进一步提升政府决策科学化、社会治理精准化、公共服务高效化水平。

　　大力实施生态建设“百千万”行动。按照城市园林化、郊区森林化的思路，着力建设“四季常青、三季有花、鸟语花香”的森林花园城市，构建具有三门峡特色的森林城市体系和绿色生态屏障。开展百里黄河湿地修复行动。用足用好政策，积极争取资金，全面启动百里黄河湿地修复工程，对核心区的108公里、21.6万亩湿地进行整体规划、全面修复，努力打造国内第一湿地长廊，把黄河湿地打造成新的“城市风景线”。开展千里城市绿廊行动。落实森林河南建设要求，加快连霍高速、三淅高速、郑西高铁、国省道两侧绿色生态廊道建设，实现绿荫成片，绿线相连，打造一批具有地域特色的靓丽工程，使我市林业生态建设工作继续走在全省前列。开展万亩矿山修复行动。按照“谁破坏、谁修复，谁所有、谁投资，谁造林、谁受益”原则，对全市2.35万亩矿山进行覆土复绿修复，把金山银山重塑为绿水青山。

　　抓好重大农田水利设施建设。围绕实施“四水同治”，加快推进七大重点水利工程，确保灵宝市弘农涧河城区至函谷关段河道治理、义马市涧河及石河生态综合整治等在建工程年内实现主体完工，城市生态水系连通、鸡湾水库、渑池县一河两沟综合治理工程年内开工，加快黄河沿岸生态水系整治提升、鸡湾调水工程前期工作进度，力争尽快具备开工条件。加快灌区续建配套与节水改造，新发展高效节水灌溉3万亩，推进小型农田水利工程达标提质，解决农田灌溉“最后一公里”问题。

　　（八）聚焦改革开放，务求外融内联和重点领域实现新突破，持续增强内生发展动力。坚定不移推进改革开放再出发，让改革之路越走越宽、开放之门越开越大。

　　继续深化改革。聚焦重点领域和关键环节，全面深化各项改革，把改革红利更多地转化为发展动力。深入推进“放管服”和“最多跑一次”改革，加快推进审批服务便民化，实现“跑一次是原则、跑多次是例外”。深化投融资体制改革，加快实施金融创新工程，完善各类金融服务平台，做大政府性融资担保平台，加大“引金入峡”和利用多层次资本市场力度，补齐金融发展短板。打好国企改革歼灭战，全面完成省定 “僵尸企业”处置后续任务，确保9月底前圆满收官。扎实推进电力、天然气等公共服务领域市场化改革，不断降低发展成本，提高资源配置效率。继续推进财税、供销社、林权等制度改革。

　　扩大开放合作。积极融入国家“一带一路”战略和郑州大都市区、洛阳副中心、大西安城市群以及黄河金三角合作区，深化开放合作，增强动力活力。积极推动煤炭、有色金属开采加工等具有比较优势的企业加快产能转移，到中亚、西非等地区建立原材料和生产基地。全面加强区域合作，深入推进与北京、西安、郑州等城市以及中南大学、东北大学等与我市产业契合度高的高等院校的战略合作，成立创新联盟，建设中关村三门峡科技城、创新科技大厦，加强中科院黄河金三角分中心、西交大国家技术转移（三门峡）中心等技术转移机构建设，促进成果转化，实现融合发展、借力发展。抢抓黄河金三角合作轮值市机遇，深入推进区域基础设施、产业发展、公共服务等合作。

　　（九）聚焦民生改善，办好民生实事、解决民生难题，让民生更好地顺应民心。始终坚持以人民为中心的发展思想，围绕群众所需所盼解难题、办实事，让广大群众共享高质量发展成果，用政府的“紧日子”换来老百姓的“好日子”。

　　充实群众获得感。密切关注就业形势变化，认真做好高校毕业生、农村转移劳动力、城镇困难人员、退役军人等重点群体就业创业，城镇新增就业4万人、新增农村劳动力转移就业9000人。持续实施全民参保计划，继续提高城乡居民基础养老金标准，全面落实困难群众大病补充保险制度，确保高龄补贴顺利发放。深化医保支付方式改革，加快推进跨省异地就医即时结算。逐步建立多主体供给、多渠道保障、租购并举的住房保障制度，着力改善低收入群体居住条件。

　　提升群众幸福感。坚持教育优先发展，继续实施学前教育三年行动计划，推进义务教育优质均衡发展，加快职业技术学院新校区建设，扎实推进社会管理学院合作办学，着力打造应用技术型高等职业教育园区。统筹推进“四医联动”，深入推进分级诊疗、家庭医生签约服务和医联体建设，加快市儿童医院、市妇幼保健院等项目进度，提高区域医疗水平。办好第二十五届黄河文化旅游节、第七届特博会，加快仰韶村遗址公园、庙底沟遗址公园暨博物馆建设，认真做好仰韶文化发现100周年纪念活动筹备工作，提高公共文化服务水平。加强公共体育服务体系建设，打造城市社区十五分钟健身圈。

　　增强群众安全感。持续深化平安三门峡建设，深入推进扫黑除恶专项斗争，强化安全生产、食品药品安全等关键领域治理，提高社会治理法治化、智能化、专业化水平，做深做实“一村一警”，健全矛盾纠纷排查化解机制，提升基层治理能力。完善重大决策社会稳定风险评估机制，加强困难群体司法援助，积极回应群众诉求，维护社会大局和谐稳定。

　　加强国防教育和国防后备力量建设，支持国防和军队改革，支持驻峡解放军、武警部队、预备役部队、民兵和人民防空建设，扎实推进军民融合，围绕创建全国双拥模范城“六连冠”，深入开展双拥共建，加强退役军人服务保障体系建设，巩固军政军民团结。支持工会、共青团、妇联、科协、侨联等人民团体更好发挥桥梁纽带作用。继续做好统计、审计、外侨、港澳、对台、史志、档案、气象、地震、民族宗教、应急管理等工作。

　　三、加强自身建设，建设人民满意的服务型政府

　　民心是最大的政治，人民满意是政府工作的最高追求。我们要以正确导向引领方向、凝聚力量，对照“五个过硬”，围绕推动政府治理能力现代化，持续营造学的氛围、严的氛围、干的氛围，加快建设人民满意的法治政府、创新政府、廉洁政府和服务型政府，以新时代新担当新作为，高水平做好政府各项工作，不辱时代使命，不负人民重托。

　　（一）强化政治建设。坚持以政治建设为统领，把学习贯彻习近平新时代中国特色社会主义思想和党的十九大精神作为首要政治任务、长期政治任务，推动学习往深里走、往实里走、往心里走，进一步树牢“四个意识”，坚定“四个自信”，坚决维护习近平总书记核心地位，坚决维护党中央权威和集中统一领导，坚决贯彻中央决策部署，坚决落实省委、省政府和市委工作要求，有令必行、有禁必止，不讲条件、不搞变通，确保各级决策部署落地生根见效。

　　（二）强化依法行政。深入推进法治政府建设，坚持依宪施政、依法行政，尊法学法守法用法，自觉运用法治思维和法治方式深化改革、推动发展、化解矛盾、维护稳定。坚持依法决策，严格执行“三重一大”决策机制，进一步提高政府决策科学化、民主化、法治化水平，确保政府决策顺民心、合民意。自觉接受人大法律监督、工作监督和政协民主监督，认真办理人大代表建议和政协委员提案，让权力在阳光下运行。

　　（三）强化担当作为。牢固树立实干成就事业的理念，坚持“五比五不比”工作导向，勇当改革发展“主攻手”，全面增强“八个本领”，在推进重点工作中积累经验，在解决重大矛盾中经受考验，在完成急难险重任务中担责担难担险，展现担当，不断增强政府治理能力和服务水平。建立健全激励约束机制和容错机制，保护和调动广大干部干事创业积极性，为担当的干部担当，对负责的干部负责，让干事的干部有想头，使成事的干部有奔头，努力开创人人尽责、个个实干、奋发向上、担当作为的政府工作新局面。

　　（四）强化作风保障。继续弘扬严和实的精神，打造过硬工作作风。严格执行中央八项规定及实施细则，发扬斗争精神，驰而不息纠正“四风”，坚决防止空喊口号的形式主义、脱离实际的官僚主义，扑下身子抓落实，多深入矛盾突出、情况复杂的地方现场办公，解决问题。大力弘扬“三股劲”精神和“干事干练干净”的务实作风，持续深化“效能革命”，树立干的导向、弘扬干的精神、倡树干的作风，以作风转变推动工作落实。深入推进政府系统党风廉政建设和反腐败工作，大力营造风清气正的良好政治生态，秉公廉洁用权，勤勉干净做事，努力创造无愧于新时代要求的新业绩。

　　各位代表！

　　征程万里风正劲，重任千钧再扬帆。让我们更加紧密地团结在以习近平同志为核心的党中央周围，高举习近平新时代中国特色社会主义思想伟大旗帜，在省委、省政府和市委的坚强领导下，同心同德，砥砺奋进，奋力谱写高质量发展新篇章，为中原更加出彩贡献强劲的三门峡力量，以优异成绩迎接中华人民共和国成立70周年！
